# Effects of naphthalene on soil fauna abundance and enzyme activity in the subalpine forest of western Sichuan, China

**DOI:** 10.1038/s41598-019-39603-6

**Published:** 2019-02-26

**Authors:** Yuwei Liu, Fan Yang, Wanqin Yang, Fuzhong Wu, Zhenfeng Xu, Yang Liu, Li Zhang, Kai Yue, Xiangyin Ni, Liying Lan, Ya Chen, Bo Tan

**Affiliations:** 1 Institute of Ecology & Forestry, Sichuan Agricultural University, Forestry Ecological Engineering in Upper Reaches of Yangtze River Key Laboratory of Sichuan Province, Alpine Forest Ecosystem Research Station, Soil and Water Conservation and Desertification Control Key Laboratory of Sichuan Province, Chengdu, 611130 China; 2 Collaborative Innovation Center of Ecological Security in the Upper Reaches of Yangtze River, Chengdu, 611130 China

## Abstract

Naphthalene has been widely used to study the role of soil fauna, but its potential non-target effects on soil enzyme activity remain unknown in subalpine forests. We added naphthalene for two years and determined the effect of such additions on the abundance of soil fauna and soil enzyme activities (β-glucosidase, cellobiohydrolase, invertase, peroxidase, polyphenol oxidase, N-acetyl-β-D-glucosaminidase, leucine arylamidase, urease, nitrate reductase and nitrite reductase) in a subalpine forest. Naphthalene could efficiently suppress the individual density and population of soil fauna *in situ*. The individual density and number of groups were decreased by 72.6–84.8% and 15.0–28.0%, respectively. Naphthalene significantly affected the activities of β-glucosidase, cellobiohydrolase, polyphenol oxidase, N-acetyl-β-D-glucosaminidase, leucine arylamidase and nitrite reductase and the activity increased in the first litter peak of naphthalene addition, and decreased at the later. The activities of β-glucosidase, cellobiohydrolase, polyphenol oxidase, peroxidase, N-acetyl-β-D-glucosaminidase, leucine arylamidase and nitrite reductase showed a negative correlation with the soil microbial PLFAs. Conversely, the activities of invertase, urease and nitrate reductase were positively correlated with the soil microbial PLFAs. Our results suggest that naphthalene is an effective method to reduce soil fauna in subalpine forest. The enzyme activity was influenced by soil fauna and microbial PLFAs.

## Introduction

Soil fauna is recognized as an indispensable component of soil biogeochemical cycling^1^. Numerous studies have demonstrated that soil fauna, such as earthworms, millipedes, springtails and mites, play an important role in accelerating the decomposition of organic matter, transforming nutrients and regulating microbial activities in various ecosystems^2–4^. Therefore, biogeochemical cycling cannot be accurately modelled without an explicit understanding of the soil fauna functions and their interactions with microorganisms in the soil detrital food web^5^.

It is challenging to research the effects of soil fauna on soil biogeochemical cycling *in situ* without affecting non-target species or changing the soil microclimate^1^. In general, physical barrier and chemical inhibition are traditional methods to assess how soil fauna can influence the litter decomposition process. The former uses bags with different mesh sizes to exclude soil fauna over a certain size (body width) from the inside litter, while the latter uses biocides to restrict the presence of soil fauna on forest floor^6^. However, there are two main disadvantages in removing soil fauna using the litter bag method. One disadvantage is that the microclimate in the litter bag might be changed due to the physical barrier presented by bags of different mesh sizes, which might further affect the activity of the soil organisms (fauna and microbes) during the decomposition processes. Differences in litter decomposition rates might therefore be attributed to differences in microclimate rather than in soil faunal structure^2^. The other disadvantage is that higher amounts of fragmented litter substrate may fall out of a large mesh bag than a small mesh bag. As a result, compared with fine mesh, soil fauna effects on litter mass loss would be overestimated for coarse mesh due to substrate decay through fragmentation and redistribution of litter by soil fauna activities and water flow^7^. Kampichler and Bruckner^7^ conducted a meta-analysis to discuss the role of microarthropods in terrestrial decomposition and the problems associated with the use of the litterbag method. These authors concluded that after 40 years of litterbag studies, our knowledge of the role of microarthropods in litter mass loss remains limited, and they suggested that it might be time to adopt another approach to answer this question.

The role of soil fauna in soil biogeochemical cycling has also been measured by suppressing certain groups of soil fauna communities through the application of biocides such as nematicides, insecticides and fungicides. Naphthalene, a polycyclic aromatic hydrocarbon (C_10_H_8_), is often used as a biocide to reduce soil and litter arthropod populations and to determine the role of soil fauna in litter decomposition processes in field experiments^1,6^. This approach showed that soil fauna can significantly improve decomposition rates. For instance^8,9^, showed that the decomposition rate of litter was decreased by 34% when arthropods were suppressed using naphthalene. Additionally^10^, indicated that the effect of the fauna was minimal at a temperate site where the fauna tended to increase the decomposition rate only towards the end of the experiment. By contrast, the effect of fauna at tropical sites was considerable within months of the start of the experiment. Similarly, a global experiment testing the influence of soil fauna demonstrated that soil fauna increases decomposition rates in temperate and wet tropical ecosystems but not in ecosystems where biological activity is constrained by climatic conditions^11^. These studies support the usefulness of naphthalene for soil fauna suppression in ecological studies.

Nevertheless, it has long been suspected that naphthalene may indirectly influence soil processes through its potential non-target effects on the microbial community and soil nutrients^1^. Early microcosm studies suggested that naphthalene might directly affect microbial populations and activity and alter nitrogen dynamics^6^. By contrast, a recent study showed that naphthalene addition is a feasible method to reduce soil micro-arthropods in the field, with negligible direct effects on soil nematodes, microbial abundance and carbon dynamics^1^. Moreover, the restriction efficiency of naphthalene on soil fauna and its non-target effects vary substantially in the field with variations in climatic conditions and soil types^1,9,12^. For example, the restriction efficiency of naphthalene on soil fauna in a sandy acrisol was higher than that in a silt–clay–loam Mollisol; naphthalene addition significantly reduced the abundance of soil micro-arthropods approximately 50% in temperate forests, while the abundance of soil micro-arthropods was reduced by 90% in tropical forests^1,13,14^. However, little is known regarding the restriction efficiency of naphthalene on soil fauna and its non-target effects in subalpine forests. Consequently, there is a clear need for a rigorous field assessment of the efficiency of naphthalene additions in suppressing soil fauna and to determine whether there are direct non-target effects on soil biochemical properties in subalpine forests.

In this study, we added naphthalene at a rate (100 g m^−2^) monthly for two years to a subalpine forest in western Sichuan and determined the effects of such additions on the abundance of soil arthropods and soil enzyme activities. The aims were (1) to estimate the efficacy of naphthalene additions on soil fauna, specifically soil arthropods, over a 24-month field incubation period and (2) to assess whether there were potential non-target effects on soil biochemical properties (enzyme activities) in the subalpine forest ecosystem. The study was designed to specifically test the following hypotheses that: (i) Naphthalene is more efficient in inhibiting meso-and microfauna because meso- & microfaunas are more sensitive to environmental changes^15^. (ii) Naphthalene addition has a greater effect on the activity of soil nitrogen-degrading enzymes than on soil carbon-degrading enzymes because, as previous studies have shown the non-target effect of naphthalene has a stronger effect on soil nitrogen turnover^6^.

## Results

### Soil fauna

**Figure 1:**
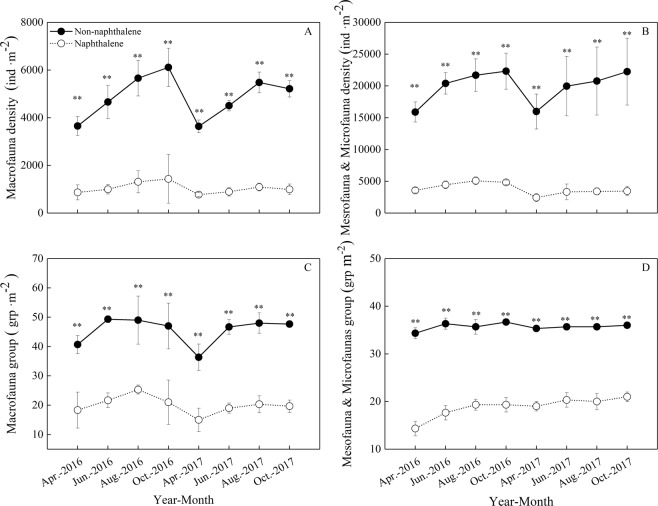
Effects of naphthalene on individual density and number of groups of soil fauna in the subalpine forest of western Sichuan. Values represent the means ± SE (n = 3, three sites with three replicates at each site). Asterisks indicate significant (*P < 0.05, **P < 0.01) differences in soil arthropod density and number of groups between different sampling times.

**Figure 2:**
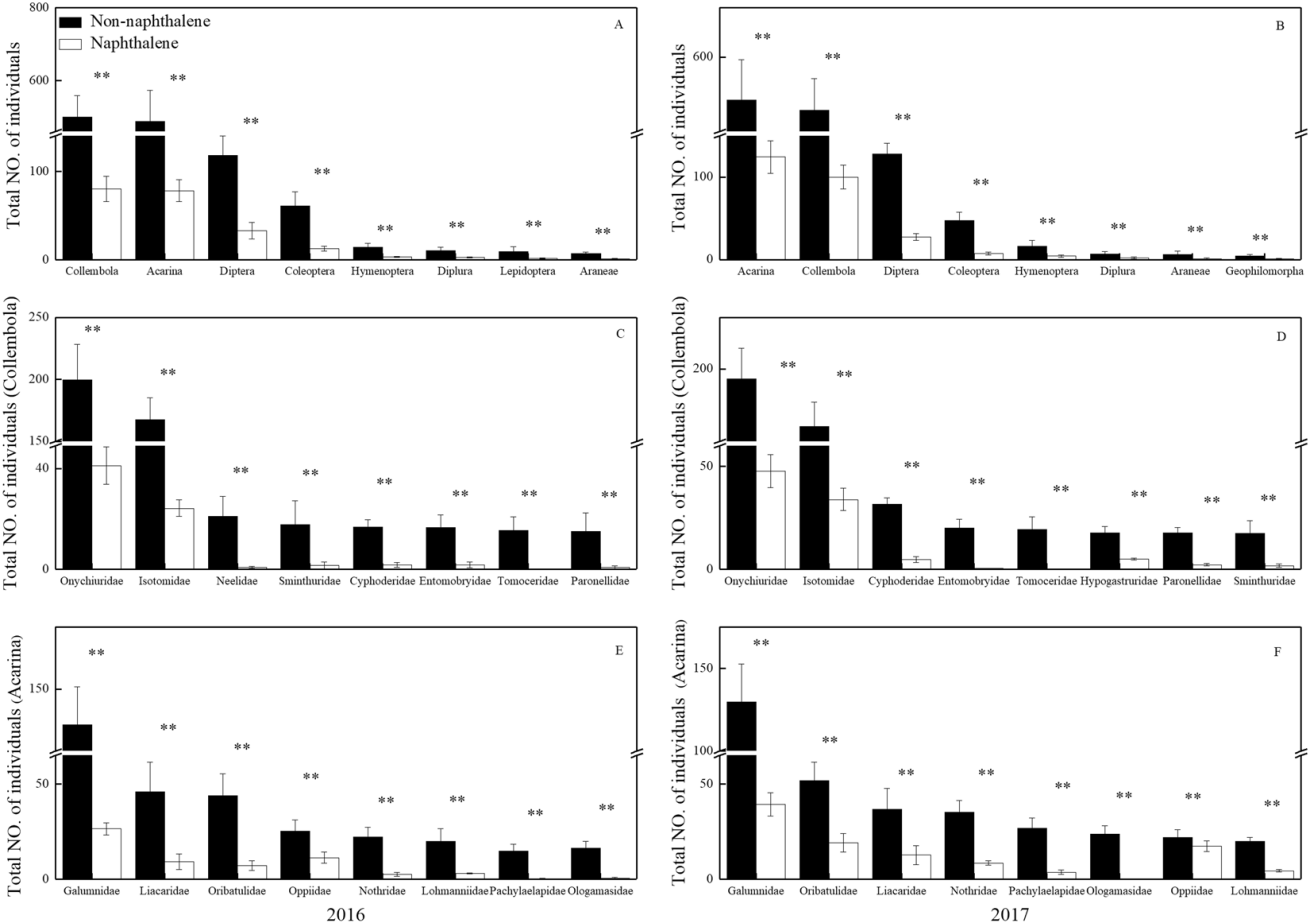
Effects of naphthalene on the number of dominant fauna in the subalpine forest of western Sichuan. (**A**) Dominant group of soil arthropods in 2016 (at the order level); (**B**) Dominant group of soil arthropods in 2017 (at the order level); (**C**) dominant group of Collembola in 2016 (at the family level); (**D**) dominant group of Collembola in 2017 (at the family level); (**E**) dominant group of Acarina in 2016 (at the family level); (**F**) dominant group of Collembola in 2017 (at the family level); Values represent the means ± SE (n = 3, three sites with three replicates at each site). Asterisks indicate significant (*P < 0.05, **P < 0.01) differences in soil arthropod density and number of groups between different sampling times.

**Figure 3:**
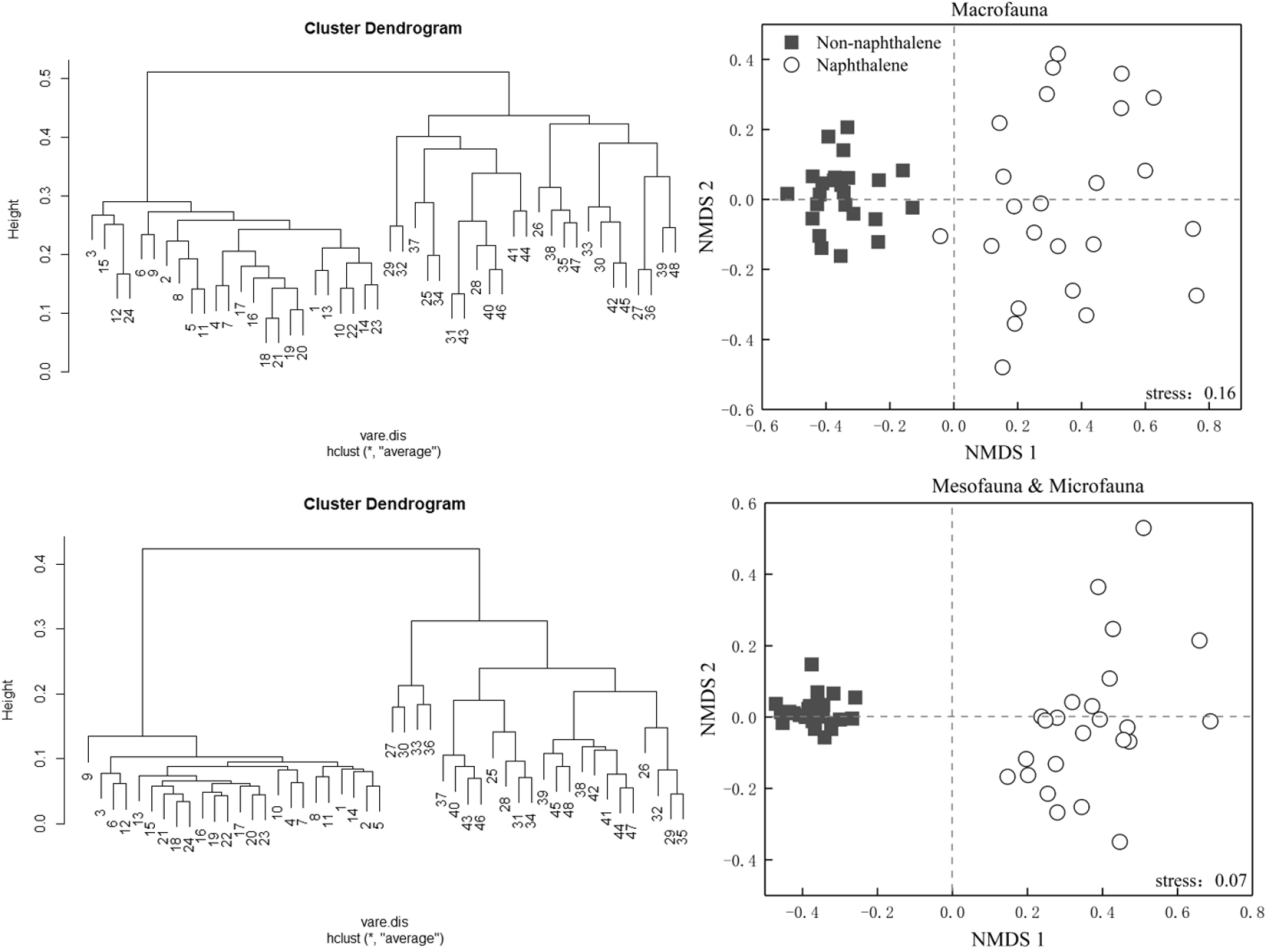
Grouping average clustering and non-metric multidimensional scaling (NMDS) of naphthalene on the macrofauna and mesofauna & microfauna in the subalpine forest of western Sichuan. 1–24: species without naphtalene; 25–48: species with naphthalene addition.

Naphthalene addition was effective in reducing the abundance of soil fauna in the subalpine forest (Fig. 1); compared to the without naphthalene addition, the individual density and species richness of macrofauna were decreased by 72.6–80.9% (*P* = 0.001) and 21.3–28.0% (*P* = 0.001), respectively. Similarly, for the meso- and microfauna, reductions of 77.4–84.8% (*P* = 0.001) in individual density and of 15.0–20.0% (*P* = 0.001) in species richness were found with naphthalene addition. Collembola and Acarina were the two numerically dominant taxa at all sites (Fig. 2AB) and represented an almost constant proportion (80.1%) of the fauna in both the naphthalene-treated and untreated sites. Naphthalene treatment reduced the individual density of Collembola and Acarina by 78.1–79.6% (*P* = 0.001). Onychiuridae (16.1%), Isotomidae (12.5%), Galumnidae (11.2%) and Liacaridae (3.5%) constituted the dominant componentsof Collembola and Acarina at the two sites (Fig. 2C–F). Saprozoic (51.0%) were the main functional group of macrofauna, whereas fungivores forms (57.6%) made up the largest functional group of meso- and microfauna (Table 1). NMDS analysis showed that stress of macrofauna was 0.16, stress of medium and small soil was 0.07. ANOSIM test showed that the naphthalene treatment had significant effect on the community structure of macrofauna (R = 0.83, *P* = 0.001) and mesofauna & microfauna (R = 0.96, *P* = 0.001) (Fig. 3).

**Table 1:** Effects of naphthalene on functional group (P: predatory; H: herbivorous; F: fungivorous forms; S: saprozoic) composition of soil arthropods under different treatments (C: control (no naphthalene); T: treatment (naphthalene)) in the subalpine forest of western Sichuan.

Functional groups	2016								2017							
	Apr.		Jun.		Aug.		Otc.		Apr.		Jun.		Aug.		Otc.	
	C	T	C	T	C	T	C	T	C	T	C	T	C	T	C	T
**Macrofaunas**																
P	953.33	166.67	1126.67	186.67	131.33	266.67	1440	266.67	713.33	153.33	1020	186.67	1240	206.67	1160	173.33
H	573.33	160	1086.67	180	1620	213.33	1260	160	366.67	113.33	840	166.67	1160	233.33	806.67	106.67
F	360	93.33	33.33	26.67	400	53.33	366.67	146.67	326.67	46.67	340	26.67	466.67	46.67	440	60
S	176.67	446.67	2113.33	606.67	2326.67	780	3046.67	860	2233.33	460	2306.67	513.33	2613.33	606.67	2806.67	653.33
**Mesofaunas & Microfaunas**																
P	4560	866.67	5300	1053.33	6506.67	906.67	5973.33	1126.67	4633.33	1233.33	6086.67	1520	6920	1740	6893.33	1453.33
H	1353.33	213.33	1960	333.33	2073.33	533.33	2853.33	466.67	1586.67	286.667	1840	360	2126.67	500	2346.67	480
F	9786.67	1293.33	12360	1873.33	11680	1926.67	12906.7	1760	9306.67	1920	11953.3	2473.33	12206.7	2693.33	12546.7	2713.33
S	273.33	53.33	366.67	73.33	526.67	46.67	533.33	113.33	400	140	573.33	93.33	460	160	580	186.67

### Activities of soil carbon-degrading enzymes

**Figure 4:**
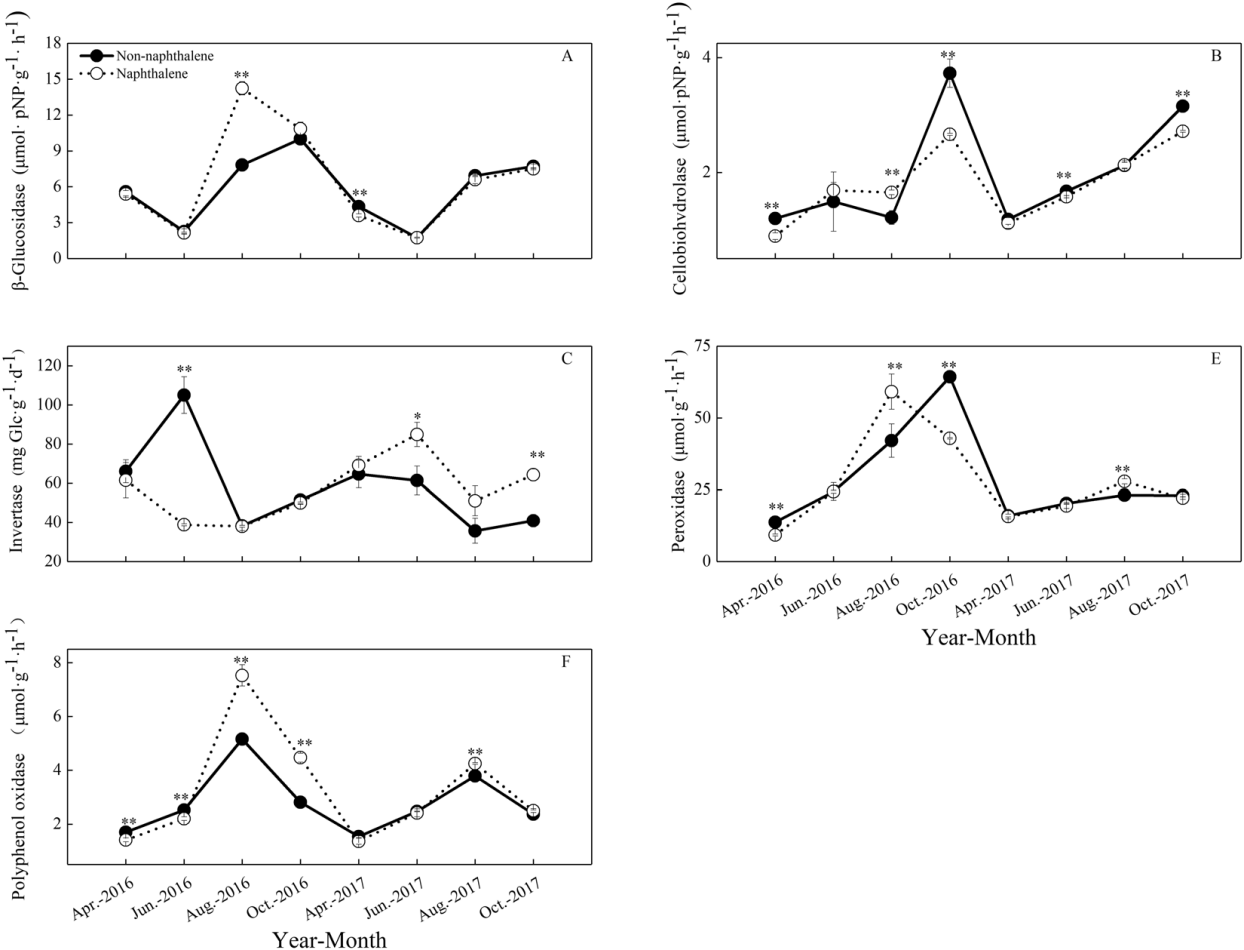
Effects of naphthalene on carbon-degrading enzymes in the subalpine forest of western Sichuan. Values represent the means ± SE (n = 3, three sites with three replicates at each site). Asterisks indicate significant (*P < 0.05, **P < 0.01.) differences in soil arthropod density and number of groups between different sampling times.

**Figure 5:**
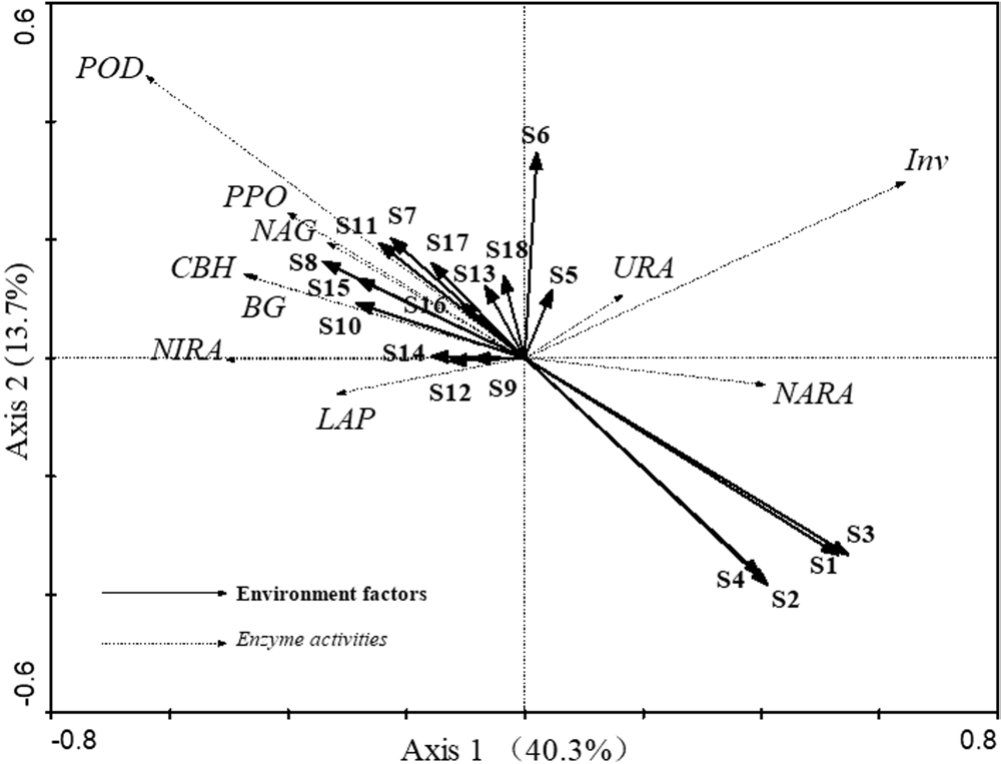
Redundancy analyses (RDA) between the soil enzyme activities and environmental factors. Inv: invertase; URA: urease activity; NARA: nitrate reductase activity; NIRA: nitrite reductase activity; S1: bacterial PLFA content; S2: fungal PLFA content; S3: Gram-positive bacteria; S4: Gram-negative bacteria; S5: the ratio of fungi to bacteria; S6: the ratio of Gram-positive bacteria to Gram-negative bacteria; S7: macrofaunas/predators; S8: macrofaunas/phytophages; S9: macrofaunas/fungivores; S10: macrofaunas/saprophytes; S11: mesofaunas and microfaunas/predators; S12: mesofaunas and microfaunas/phytophages; S13: mesofaunas and microfaunas/fungivores; S14: mesofaunas and microfaunas/saprophytes; S15: macrofauna density; S16: mesofauna and microfauna density; S17: macrofauna groups; S18: mesofauna and microfauna groups.

During the entire study period, similar dynamics of soil carbon-degrading enzymes were exhibited in both the naphthalene-treated and untreated sites (Fig. 4), and the naphthalene significantly altered the activities of β-glucosidase (BG), cellobiohydrolase (CBH) and polyphenol oxidase (PPO) over time (Table 2). The naphthalene addition obviously increased the BG (*P* = 0.001), CBH (*P* = 0.004), PPO (*P* = 0.001) and peroxidase (POD) (*P* = 0.025) activities in August 2016, but it dramatically decreased the PPO (*P* = 0.004) and POD (*P* = 0.004) activities in April 2016. In the RDA (Fig. 5), the soil microbial PLFAs and soil fauna formed the first two-dimensional axis of the multiple factor analysis and accounted for 40.3% and 13.3%, respectively, of the total variance. G^+^ PLFAs (13.6%, *P* = 0.004) were the most significant explanatory variable for enzyme activities, followed by bacterial PLFAs (12.9%, *P* = 0.008), fungal PLFAs (8.9%, *P* = 0.018) and G^−^ PLFAs (8.3%, *P* = 0.022). Moreover, the activities of BG and CBH showed a negative correlation with the soil microbial PLFAs, while the activities of invertase (Inv), PPO and POD showed a positive correlation (Fig. 5).

**Table 2:** Results of Repeated-measures ANOVAs, which tested the effects of naphthalene treatment (N) and sampling time (T) and their interaction on soil enzyme activities (BG: β - Glucosidase; CBH: Cellobiohydrolase; NAG: N – Acetyl – β – D - glucosaminidase; LAP: Leucine arylamidase; POD: Peroxidase; PPO: Polyphenol oxidase) in the subalpine forest of western Sichuan.

Variable	N			T			N × T		
	*df*	*F*	*P*	*df*	*F*	*P*	*df*	*F*	*P*
BG	1	50.281	0.002	7	1096.067	0.000	7	129.713	0.000
CBH	1	54.025	0.002	7	139.619	0.000	7	11.268	0.000
Invertase	1	0.079	0.793	7	44.798	0.000	7	52.767	0.000
POD	1	1.902	0.240	7	250.353	0.000	7	28.962	0.000
PPO	1	186.718	0.000	7	773.361	0.000	7	74.576	0.000
NAG	1	71.779	0.001	7	267.628	0.000	7	6.139	0.022
LAP	1	27.644	0.000	7	390.411	0.000	7	12.980	0.000
Urease	1	3.1828	0.122	7	199.460	0.000	7	20.111	0.000
Nitrate reductase	1	6.770	0.060	7	143.760	0.000	7	9.662	0.000
Nitrite reductase	1	8.984	0.040	7	114.363	0.000	7	2.026	0.056

### Activities of soil nitrogen-degrading enzymes

**Figure 6:**
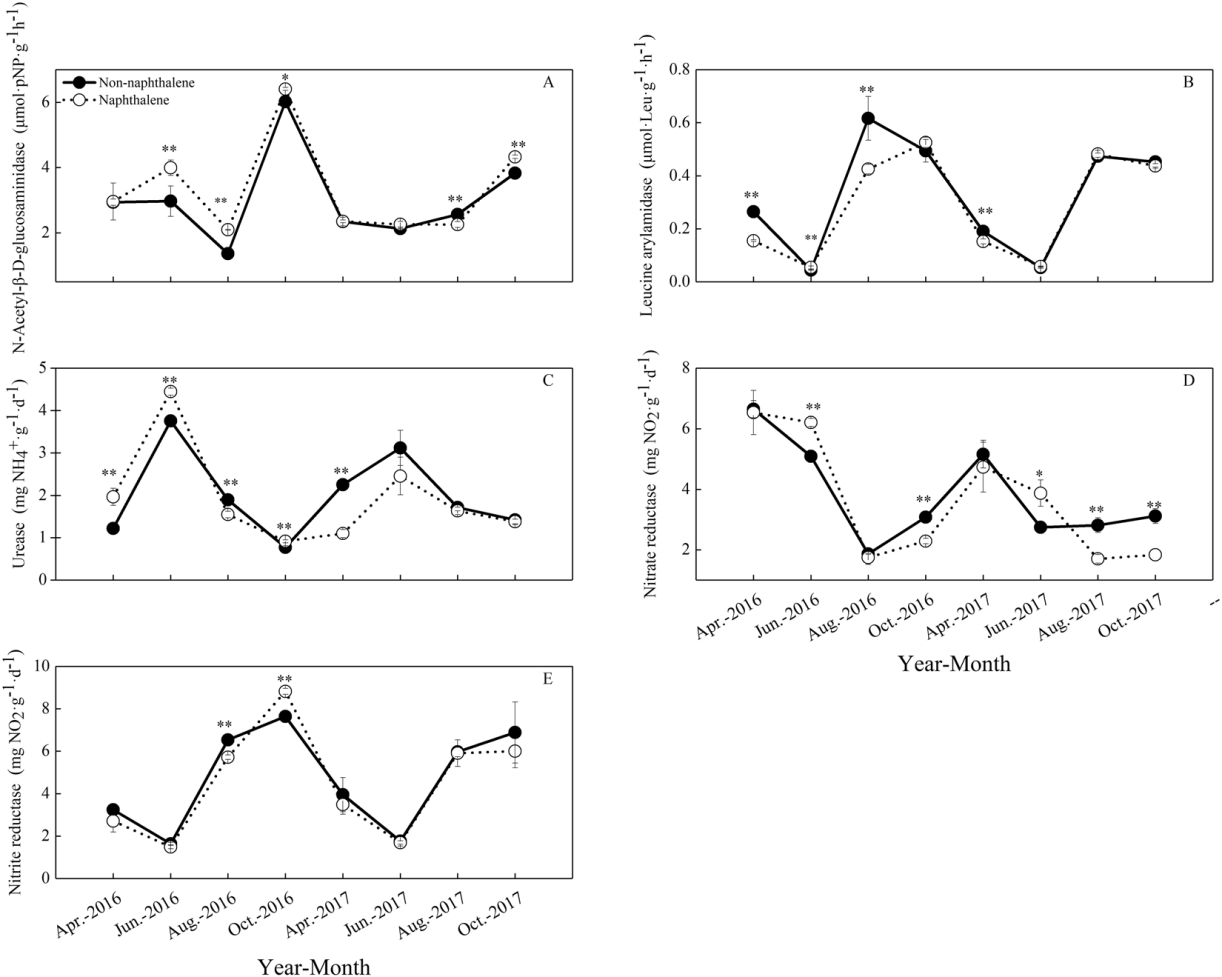
Effects of naphthalene on nitrogen-degrading enzymes in the subalpine forest of western Sichuan. Values represent the means ± SE (n = 3, three sites with three replicates at each site). Asterisks indicate significant (*P < 0.05, **P < 0.01.) differences in soil arthropod density and number of groups between different sampling times.

During the entire study period, similar dynamics of soil nitrogen-degrading enzymes were observed in both the naphthalene-teated and untreated sites (Fig. 6). Naphthalene had a significant effect on N-acetyl-β-D-glucosaminidase (NAG) (*P* = 0.000), leucine arylamidase (LAP) (*P* = 0.001) and nitrite reductase (NIRA) (*P* = 0.040) activities, but it had insignificant effect little influence on urease (URA) (*P* = 0.122) and nitrate reductase (NARA) (*P* = 0.060) activities over time (Table 2). The naphthalene treatment significantly increased NAG (*P* = 0.027), URA (*P* = 0.000) and NARA (*P* = 0.000) activities in June 2016, but it dramatically decreased LAP (*P* = 0.017), URA (*P* = 0.006) and NIRA (*P* = 0.001) activities in August 2016. The RDA results indicated that URA and NARA were positively affected by the microbial community structure (Fig. 5). Conversely, NAG, LAP and NIRA were negatively correlated with the soil microbial PLFAs (Fig. 5).

## Discussion

### Naphthalene effects on soil fauna

Confirming our first hypothesis, the addtition of naphthalene to the soil surface highly and significantly reduced soil arthropod abundance; at the naphthalene-treated sites, the individual densities of macrofauna and meso- and microfauna decreased by 72.6–80.9% and 77.4–84.8%, respectively, and the species richness of macrofauna and meso- and microfauna decreased by 21.3–28% and 15.0–20.0%, respectively. Moreover, the inhibitive efficiency of naphthalene does not change with time, which means that soil arthropods do not adapt to the presence of naphthalene. This finding is consistent with Cotrufo’s findings^1^. The efficacy of naphthalene additions with soil macrofauna were all excluded and total microarthropods also reduced by over 60 percent in evergreen broadleaf forest, coniferous forest, dwarf forest and alpine meadow^12^. The efficacy of naphthalene additions with respect to soil meso- and microfauna in our subalpine forest was lower than that in a litterbag study of temperate subalpine forest^9^. These differences with our study are due to the combination of the physical barrier of the litterbags and the chemical inhibition of naphthalene additions, which might enhance the inhibition efficiency relating to the soil faunal community. However, the efficacy of naphthalene additions with respect to soil meso- and microfauna in our study was higher than that in a tall grass prairie ecosystem (45–52%)^1^. The differences in inhibition efficiency are due to variations in the amount and frequency of naphthalene application, the interval of sampling periods and the depth of soil sampling.

Although the concentration and frequency of naphthalene additions did not exclude all macrofauna and meso- and microfauna in our experiment, the total number of soil arthropods decreased by more than 70% in the naphthalene treatment plots. In particular, individuals of Collembola and Acarina, the two numerically dominant taxa, were reduced by approximately 80% over the entire study period. Collembola and Acarina have been reported to play an important role in carbon and nutrient transformation, storage and release during litter decomposition^16,17^. These two suppressed groups consisted of more than 80% fungivorous and predator feeders, and a reduction in their abundance might influence microbial abundance and structure by changing the microbial grazing pressure related to predatory mites and springtails. Moreover, Peng *et al*.^18^ found a positive relationship between the richness of soil arthropods and litter decomposition rates, and changes in the abundance and structure of some faunal functional groups or the composition of microbe-consuming species might exert considerable effects on litter decomposition and nutrient cycling. This experiment suggests that naphthalene addition is a feasible method to reduce soil arthropods in subalpine forest ecosystems of western Sichuan.

### Naphthalene effects on soil enzymes

For decades, the use of naphthalene to eliminate groups of soil fauna in field studies has been avoided due to its potential non-target effects on other soil organisms. Generally, naphthalene additions may affect extracellular enzyme activity in two ways^1^. First, reducing or removing targeted soil organism groups may directly affect other groups by altering the interactions among these groups. For instance, removing microarthropods could affect extracellular enzyme activity if the species-specific trophic behaviours of microarthropods-microbes significantly influence microbial activity or abundance^19^. Second, the application of naphthalene might cause a non-target effect on soil enzyme activity and nutrient cycling processes by stimulating soil respiration, microbial immobilization and the number of bacteria^6^. For example, naphthalene can be used as a substrate for microbial carbon, changing microbial biomass and community composition, and CO_2_ emissions, thus affecting the entire soil carbon cycle^6^.

Previous studies demonstrated that soil fauna could participate in the transformation and degradation of soil and litter organic matter through the action of feeding, crushing and intestinal digestion enzymatic degradation^20^. For example, earthworm activity significantly increased the activities of β-glucosidase and cellobiose hydrolase in soil and promoted the decomposition of carbohydrates^21^, and soil arthropods significantly increased cellulase activity during the litter decomposition process of a tropical forest^22^. Meanwhile, soil fauna can also influence the synthesis, secretion and activity of soil enzymes through selective feeding of microbes, inoculation of propagating microbes, adjustment of microbial community structure, and other indirect effects on the synthesis, secretion and activity of soil enzymes^20,23^. For instance, the presence of bacterial-feeding protists and nematodes changed the relative abundance of dominant bacterial populations^24^, and the presence of these soil fauna significantly increased soil microbial basal respiration and microbial turnover, measured as the microbial metabolic quotient^20^. In this study, the soil microbial PLFAs and soil fauna structure explained more than half (53.6%) of the total variance in extracellular enzyme activity and showed a significant correlation with the enzyme activity, which is consistent with previous studies^25,26^.

In contrast to the second hypothesis, both soil carbon-degrading enzymes and nitrogen-degrading enzymes were obviously influenced by naphthalene addition in the subalpine forest. BG and CBH are enzymes involved in the degradation of cellulose, and their activities related to cellulose degradation are significantly affected by soil fauna^27^ and soil microbial biomass^28^. Soil microarthropods (mainly Collembola and mites) play an important role in soil carbon dynamics, and they can partly feed on nematodes, arthropods or their eggs^29^. Moreover, the predatory and fungal soil fauna can stimulate the growth of microorganisms and increase enzyme activity^29^. The suppressed individuals of fungivorous and predator microarthropods in this experiment may have reduced microbial grazing pressure as a result of the decrease in predatory microarthropods. Therefore, naphthalene additions decreased the activities of BG and CBH.

POD and PPO are involved in the degradation of hydrocarbons and their metabolic intermediates. In our study, the POD activity was not affected by naphthalene treatment, which may be due to the formation of peroxidase in the biochemical oxidation reaction of biological respiration and organic matter. Several studies^6,30,31^ have shown that naphthalene treatment did not significantly alter the soil respiration rate; thus, naphthalene treatment did not have an impact on peroxidase activity. Polyphenol oxidase activity is usually related to microbial community structure^2^. Studies have shown that naphthalene can be used as a substrate for microbial carbon, resulting in a change in the microbial biomass and microbial community^1^. In addition, a study showed that the activity of polyphenol oxidase is related to soil fauna^32^. Mora *et al*.^32^ found that the activity of polyphenol oxidase was significantly related to biological factors, especially wormcasts, in a neotropical savannah. Therefore, naphthalene treatment reduced the numbers and groups of soil fauna and changed the microbial community, which was the main reason for the difference in polyphenol oxidase enzyme activity.

NAG and LAP are the key enzymes involved in soil nitrogen cycling. NAG is a chitin-degrading enzyme that is also involved in the degradation of peptidoglycan, which is a component of the bacterial cell wall. LAP can break the N-terminal amino acid of the polypeptide into leucine^33,34^. Amino acids and other nitrogenous compounds are important forms of soluble soil organic carbon^35^; thus, their activities are closely related to microbial available nutrients. Blair *et al*.^6^ conducted an experiment on the effect of naphthalene on nitrogen pools of litter and showed that naphthalene could decrease the final concentration and absolute content of nitrogen, as well as soil extractable NH_4_-N and NO_3_-N + NO_2_-N. Therefore, the activities of NAG and LAP were significantly affected by naphthalene treatment during the study period. Additionally, urease, nitrate reductase and nitrite reductase are important participants in the process of soil nitrification and denitrification. Urease^36^ is an important hydrolytic biological enzyme for the conversion of organic nitrogen to available nitrogen. Nitrate reductase and nitrite reductase transform soil available nitrogen into non-effective molecular nitrogen; in addition, their activity is affected by available nitrogen, such as NH_4_^+^ and NO_3_^−^ in soil^37^. Cole *et al*.^38^ found that single species and variations in the diversity of microarthropods influenced soil microbial abundance in a British grassland; however, there was no effect on microbial or plant uptake of nitrogen. Therefore, urease and nitrate reductase were not affected in this study. The effect of nitrite reductase may be that it is more closely related to soil fauna and is more affected by soil structure aeration.

## Conclusion

It is important to study the function of soil fauna to estimate the role of soil biogeochemical processes, such as plant litter decomposition. Our results demonstrated that naphthalene had different effects on the soil arthropods and soil enzymes in a subalpine forest soil in western Sichuan. Naphthalene treatment significantly inhibited the individual density and number of groups of soil fauna, i.e., by 72.6–84.8% and 15.0–28%, respectively. Naphthalene significantly affected the activities of BG, CBH, PPO, NAG, LAP and nitrite reductase. However, there was no significant effect on the activities of POD, urease, nitrate reductase. The relationship between microbial community structure (G^+^ PLFAs, bacterial PLFAs, fungal PLFAs and G^−^ PLFAs) and enzyme activity was significant. Therefore, naphthalene addition is a feasible method to reduce soil arthropods in subalpine forest ecosystems of western Sichuan. These results provide basic data for a further understanding of the interaction between soil fauna and microorganisms involved in key ecological processes, such as material cycling and nutrient utilization, in subalpine forests in Western Sichuan Province.

## Materials and Methods

### Study site

This study was conducted at the Long-term Research Station of Alpine Forest Ecosystems, which is located on the eastern Tibetan Plateau, China (31°18′N, 102°56′E, 3023 m *a.s.l*). The study site is a secondary fir (*Abies faxoniana*) forest formed by the seeding of areas that had been clear-cut in the 1960s. The mean annual air temperature and the mean annual rainfall at the site are 2.7 °C and 850 mm, respectively^39^. The soil temperature decreases below 0 °C and remains frozen during the entire cold snow season from late November to late March^40^. The tree canopy is dominated by 60-year-old *Abies faxoniana*, and the tree canopy coverage is 0.7. The average tree height and DBH are 17 m and 24 cm, respectively. The understory shrubs are dominated by *Salix paraplesia*, *Fargesia nitida*, *Rhododendron lapponicum*, *Berberis sargentiana*, *Sorbus rufopilosa, Rosa sweginzowii* and *Hippophae rhamnoides* and other species. The herb layer is dominated by *Cacalia auriculata*, *Cystopteris montana*, *Carex spp*., *Cyperus spp*. and other species. The soil is classified as a Cambic Umbrisol according to the IUSS Working Group, and the basic chemical soil properties (0–15 cm) are as follows: pH 6.5 ± 0.3, total organic carbon 153.9 ± 27.4 g kg^−1^, total nitrogen 7.8 ± 1.3 g kg^−1^ and phosphorus 0.9 ± 0.1 g kg^−1^ ^41^.

### Experimental design

The naphthalene manipulation experiment consisted of a control without naphthalene and naphthalene treatment. Each treatment had five plots or replicates (5 m × 5 m each) spaced ≥10 m apart. Within each plot, there were four subplots (2 m × 2 m each), two of which were treated with naphthalene, while the others served as a control.

For each subplot, a PVC fence (2 m × 2 m × 10 cm) was inserted in the ground to 5 cm depth. The naphthalene addition started in early October 2015, and the naphthalene treatment subplots received the equivalent of 100 g m^−2^ naphthalene^9,14^ every month for the full duration of the experiment.

### Soil sampling

A total of eight sampling events were performed, on (1) 19 April 2016, (2) 15 June 2016, (3) 18 August 2016, (4) 23 October 2016, (5) 19 April 2017, (6) 15 June 2017, (7) 18 August 2017 and (8) 23 October 2017. On each sampling date, an intact soil core (20 × 25 cm) was collected to a depth of 10 cm for soil arthropod extraction. Moreover, five soil cores (100 g each) from each subplot were collected using a soil auger (15 cm depth and 5 cm diameter) and mixed as one composite sample after the removal of visible debris and fresh litter. Soil samples were stored in freezer boxes and transported to the laboratory within 24 h. Fresh soils were passed through a 2.0-mm sieve and then stored in a refrigerator at 4 °C for less than one week for microbial and chemical analysis.

#### Fauna extraction

Faunas were extracted from the intact soil core by the Tullgren funnel (mesh size 4.00 mm) method over a period of 48 h. All collected soil faunas were enumerated and classified by microscope analysis. Most of the collected soil fauna were identified to the family level following the key protocols of^42^. On the date of fauna extraction, approximately 20 g of fresh soil was weighed and oven dried at 105 °C and used to calculate the gravimetric soil water content and for soil dry-weight correction. Soil faunas were classified into macrofauna (>2 mm) and meso- and microfauna (0.1–2.0 mm) based on body size. According to the relevant literature, the collected soil fauna was divided into four functional groups according to their feeding characteristics: predatory, herbivorous, saprozoic and fungivorous forms^43,44^.

#### Microorganism PLFAs

Soil microbial biomass was estimated using phospholipid fatty acids (PLFAs), which were extracted and quantified using a modified method described by^45^. Briefly, total lipids were extracted from 2 g of fresh soil according to a one-phase extraction technique using phosphate buffer, methanol and chloroform in a 0.8:2:1 (v/v/v) ratio^46^. After the addition of an internal standard (19:0), PLFAs were converted to fatty acid methyl esters (FAMEs) by alkaline methanolysis and were quantified by a gas chromatograph (GCMS-QP2010 Series, Shimadzu, Japan). The sum of all subsequently described PLFAs was used as a proxy for total microbial biomass. We used the sum of i15:0, a15:0, i16:0, i17:0 and a17:0 as Gram-positive bacterial markers^47,48^; 16:1ω7c, 16:1ω9c, cy17:0, 18:1ω7c, and cy19:0 as Gram-negative bacterial markers^49^ and 15:0, 16:0, 16:1ω5t, 17:0, 18:00 and 20:5 as general bacterial markers^46,50^. Gram-positive, Gram-negative and general bacterial markers were summed to give the total bacteria. We used the sum of 18:3, 18:1ω9c, 18:2ω6, 9c and 20:1ω9c as fungal markers to represent the total fungi^27,51^. The GC-MS conditions were as follows: no shunt injection; injection port temperature of 300 °C; initial temperature of 60 °C, maintained for 1 min; temperature program: 30 °C min^−1^ increase until 150 °C, maintained for 4 min; followed by an increase to 250 °C at a rate of 4 °C min^−1^ and maintained for 15 min. Finally, the temperature was increased by 25 °C min^−1^ to 300 °C and maintained for 6 min. The interface temperature was 280 °C, helium was used as a carrier gas, and the flow rate was 0.8 mL min^−1^.

#### Soil enzyme activity analysis

Potential extracellular enzyme activities of β-glucosidase (BG), cellobiohydrolase (CBH), and N-acetyl-β-D-glucosaminidase (NAG) were measured using analogue para-nitrophenyl-β-D-glucopyranoside (pNPG) substrate. The potential activities of peroxidase (POD) and polyphenol oxidase (PPO) were assayed using L - β -3, 4-dihydroxy-phenylalanie (L-DOPA) substrate, and the potential extracellular enzyme activities of leucine arylamidase (LAP) were determined using L-leucine-p-nitroanilide substrate according a modified method described by the Bob Sinsabaugh Laboratory^27^.

Enzyme assays were conducted in black 96-well microplates. In brief, soil slurries were prepared by mixing 3 g of soil with 40 mL sodium acetate buffer (pH = 5.0) using a blender for 2 minutes. Each plate required a set of wells for the blank (empty wells or 200 μL buffer only) and the substrate control (substrate + buffer). Each sample required a set of wells for homogenate controls (homogenate + buffer) and the actual assay (homogenate + substrate). BG was incubated for 1.5 h at 30 °C, PPO and POD were incubated for 2 h at 20 °C, and NAG, CBH and LAP were incubated for 4 h at 30 °C. Then, the cells were cultured in a constant temperature incubator with a DNM-9602 enzyme marker analyser (Beijing) for detection. BG, CBH and NAG activities were expressed as μmol pNP g^−1^ dry soil d^−1^; POD and PPO activities were expressed as μmol g^−1^ dry soil d^−1^; and LAP activities were expressed as μmol Leu g^−1^ dry soil d^−1^.

In addition, analysis of soil invertase activity (mg glucose g^−1^ soil DW d^−1^) or soil urease activity (mg NH_4_^+^ g^−1^ soil DW d^−1^) was performed according to Li *et al*.^52^. Analysis of soil nitrate reductase and nitrite reductase activities (mg NO_2_ g^−1^ soil DW d^−1^) were performed according to Xiong *et al*.^53^.

### Statistical analysis

Independent t-test were used to determine the difference between the naphthalene addition and without naphthalene with soil enzyme activities and soil fauna abundance in every month; the repeated - measures ANOVAs was used to examine the effects of naphthalene and the time and the interaction of variables. Independent t-test and the repeated – measures ANOVAs were performed using IBM SPSS Statistics 20.0, and the graphs were produced using Origin PRO 2017 software.

Non-metric multidimensional scaling (NMDS) was utilized to examine community composition of macrofauna and mesofauna & microfauna. NMDS ordination uses the Bray -Curtis coefficient to measure the dissimilarity in species compositions among naphthalene addition and without naphthalene. Analysis of similarities (ANOSIM) was used to test the significance of community composition between naphthalene treatment and control. The NMDS and ANOSIM tests were performed using vegan package and MASS package of R software (version 3.5.2), and the graphs were produced using Origin PRO 2017 software.

Moreover, redundancy analysis (RDA) was used to visualize the correlations between soil enzyme activities and PLFAs, soil fauna density and soil fauna functional groups (e.g., bacterial PLFAS, fungal PLFAs, Gram-positive PLFAs, Gram-negative PLFAs) using CANOCO software (version 4.5, Microcomputer Power, Inc., Ithaca, NY). The RDA tests were considered significant at *P* < 0.05.

## Supplementary information

Effects of naphthalene on soil fauna abundance and enzyme activity in the subalpine forest of western Sichuan, China

## Data Availability

All data generated or analysed during this study are included in this published article.
