# Supplementary material for: Effects of naphthalene on soil fauna abundance and enzyme activity in the subalpine forest of western Sichuan, China
Source: Sci Rep. 2019 Feb 26;9:2849. doi: 10.1038/s41598-019-39603-6 (PMC6391496; doi:10.1038/s41598-019-39603-6)
Supplement: Supplementary file 1 — Effects of naphthalene on soil fauna abundance and enzyme activity in the subalpine forest of western Sichuan, China [file 41598_2019_39603_MOESM1_ESM.pdf]

Title:

Effects of naphthalene on soil fauna abundance and enzyme activity in the subalpine forest of western Sichuan, China

Authors:

Yuwei Liu [1,2], Fan Yang [1,2], Wanqin Yang [1,2], Fuzhong Wu [1,2], Zhenfeng Xu [1,2], Yang Liu [1,2], Li Zhang [1,2], Kai Yue [1,2], Xiangyin Ni [1,2], Liying Lan [1,2], Ya Chen [1,2] & Bo Tan [1,2]

Affiliation:

[1] Institute of Ecology & Forestry, Sichuan Agricultural University, Forestry Ecological Engineering in Upper Reaches of Yangtze River Key Laboratory of Sichuan Province, Alpine Forest Ecosystem Research Station, Soil and Water Conservation and Desertification Control Key Laboratory of Sichuan Province, Chengdu 611130, China

[2] Collaborative Innovation Center of Ecological Security in the Upper Reaches of Yangtze River, Chengdu 611130, China

Yuwei Liu and Fan Yang contributed equally to this work.

Email addresses:

liuyuweisicau@163.com; yangfansicau@163.com; scyangwq@163.com; wufzchina@163.com; xuzf@sicau.edu.cn; sicauliuyang@163.com; 692132748@qq.com; 731738583@qq.com; 574465944@qq.com; 1174749054@qq.com; bobotan1984@163.com

Corresponding authors (*):

B. Tan (E-mail address: bobotan1984@163.com),

Tel.: 86-28-86290957.

Postal address: 211 Huimin Road, Wenjiang, Chengdu 611130, Sichuan, China

Table S1 Community structure of soil fauna in the subalpine forest of western Sichuan. P: predatory; H: herbivorous; F: fungivorous forms; S: saprozoic

Species	Density of 2016 (ind m^{-2})								Density of 2017 (ind m^{-2})								Functional groups
	Apr.		Jun.		Aug.		Oct.		Apr.		Jun.		Aug.		Oct.		
	C	T	C	T	C	T	C	T	C	T	C	T	C	T	C	T	
Macrofauna																	
Lycosidae	13.33	0	20	0	20	6.67	26.67	0	0	0	10	0	20	6.67	10	6.67	P
Liocranidae	33.33	0	40.	0	53.33	0	46.67	0	5	0	30	6.67	35	0	10	0	P
Araneidae	13.33	0	40	0	20	0	0	0	0	0	30	0	30	0	5	0	P
Linyphiidae	0	0	13.33	0	13.33	0	13.33	0	0	0	10	0	10	0	10	0	P
Salticidae	33.33	13.33	40	6.67	33.33	26.67	73.33	26.67	20	0	55	26.67	70	33.33	50	6.67	P
Theridiosomatida	6.67	0	6.67	0	6.67	0	6.67	0	/	/	/	/	/	/	/	/	P
Phalangiidae	20	6.67	26.67	0	6.67	6.67	33.33	13.33	15	6.67	40	13.33	60	6.67	45	13.33	P
Neobisiidae	6.67	0	26.67	13.33	26.67	13.33	33.33	0	0	0	20	6.67	35	13.33	20	0	P
Chthonidae	20	6.67	26.67	0	46.67	6.67	66.67	0	10	0	55	6.67	60	20	60	0	P
Spirostreptidae	66.67	0	106.67	0	140	13.33	140	0	15	0	65	0	95	13.33	65	0	H
Cambalopsidae	40	6.67	53.33	6.67	86.67	26.67	40	13.33	/	/	/	/	/	/	/	/	H
Lithobiomorpha	33.33	0	66.67	0	106.67	0	60	0	20	0	85	26.67	70	6.67	60	6.67	P
Geophilomorpha	120	6.67	113.33	13.33	73.33	26.67	53.33	6.67	35	0	110	13.33	75	26.67	45	0	P
Scutigerellidae	20	0	20	26.67	40	4	0	0	15	0	40	26.67	65	20	20	13.33	P
Projapygidae	120	53.33	226.67	60	300	53.33	173.33	40	60	40	140	40	170	73.33	115	26.67	H
Microphysidae	0	0	6.67	6.67	33.33	0	13.33	0	0	0	10	6.67	5	6.67	5	0	H
Miridae	0	0	13.33	0	13.33	0	0	0	0	0	5	0	5	0	0	0	H
Sphaeropsocidae	13.33	0	120	13.33	220	13.33	133.33	20	15	0	60	13.33	70	13.33	30	0	H
Phlaeothripidae	0	0	33.33	0	46.67	0	6.67	0	0	0	15	0	35	0	0	0	H
Jassidae	33.33	0	20	6.67	26.67	6.67	60	0	40	13.33	10	0	0	0	85	13.33	H
Formicidae	193.33	80	226.67	53.33	413.33	60	280	53.33	130	60	275	106.67	395	120	270	66.67	H
Noctuidae	40	20	106.67	26.67	160	26.67	246.67	33.33	10	0	10	0	0	0	15	0	H
Geometridae	46.67	6.67	46.67	6.67	13.33	0	93.33	13.33	30	13.3	25	0	0	0	105	13.33	S
Histeridae	20	6.67	20	6.67	40	0	46.67	0	30	6.67	40	6.67	50	0	75	13.33	P
Canthaidae	26.67	6.67	33.33	0	26.67	20	60	13.33	10	0	30	6.67	45	6.67	30	6. 67	F
Anisotomidae	26.67	6.67	6.67	6.67	33.33	13.33	33.33	0	20	6.67	0	6.67	15	0	25	6.67	S
Nitidulidae	73.33	60	93.33	40	140	93.33	200	73.33	70	6.67	110	46.67	180	40	100	46.67	S
Tenebrionidae	6.67	0	13.33	6.67	20	13.33	20	0	5	0	20	0	30	6.67	5	0	H
Melandryidae	46.67	0	100	0	93.33	0	100	0	25	0	45	0	80	0	35	0	H
Scaphidiidae	6.67	6.67	13.33	13.33	20	13.33	20	6.67	10	6.67	5	6.67	20	6.67	10	6.67	P
Ptiliidae	13.33	0	33.33	6.67	80	0	40	6.67	25	0	65	6.67	55	0	35	6.67	F
Scydmaenidae	126.67	13.33	93.33	33.33	160	6.67	173.33	66.67	190	53.33	55	13.33	60	6. 67	265	53.33	P
Curculionidae	0	0	20	0	26.67	0	6.67	0	0	0	0	0	10	0	5	0	H
Pselaphidae	0	0	6.67	0	20	0	13.33	0	/	/	/	/	/	/	/	/	S
Staphylinidae	480	106.67	560	73.33	646.67	120	786.67	146.67	225	80	245	33.33	320	60	255	53.33	P
Cryptophagidae	13.33	6.67	26.67	0	33.33	6.67	6.67	0	0	0	10	0	15	0	10	0	F
Scarabaeidae	13.33	0	40	0	40	0	40	0	0	0	15	0	25	0	10	0	H
Corylophidae	0	0	0	0	20	0	6.67	0	/	/	/	/	/	/	/	/	S
Hesperinidae	440	86.67	366.67	93.33	513.33	80	833.33	180	440	106.67	320	93.33	355	160	640	193.33	S
Pachyneuridae	53.33	20	20	13.33	40	13.33	40	20	25	0	5	6. 67	15	6.67	50	0	S
Ceroplatidae	13.33	6.67	26.67	20	40	13.33	40	20	35	6.67	15	0	5	6.67	35	13.33	S
Scatopsidae	26.67	0	20	13.33	46.67	13.33	13.33	0	15	0	15	0	45	6.67	25	6.67	S
Bilionidae	106.67	46.67	186.67	53.33	233.33	66.67	140	80	185	53.33	285	106.67	295	100	135	53.33	S
Anisopodidae	6.67	0	40	6.67	13.33	0	20	0	10	0	10	0	5	0	30	0	S
Cecidomyiidae	6.67	0	40	0	66.67	6.67	60	0	5	0	25	0	30	6.67	20	0	S

continuation sheet

Limoniidae	6.67	6.67	6.67	6.67	13.33	0	6.67	13.33	30	6.67	30	6.67	25	0	40	6.67	S
Cylindrotomidae	0	0	26.67	0	40	13.33	40	6.67	15	6.67	25	0	55	0	35	6.67	S
Tipulidae	20	0	33.33	0	53.33	0	60	0	20	0	20	0	35	0	20	0	S
Trichoceridae	6.67	0	13.33	0	6.67	0	33.33	6.67	10	0	0	0	0	0	40	0	S
Mycetophilidae	66.67	26.67	53.33	40	33.33	86.67	60	86.67	85	33.33	95	20	90	13.33	80	26.67	S
Sciaridae	433.33	113.33	600	166.67	500	220	720	193.33	445	146.67	510	126.67	485	146.67	510	166.67	S
Dolichopdidae	60	6.67	33.33	6.67	20	0	46.67	13.33	15	0	25	0	25	0	40	0	F
Psychodidae	46.67	13.33	20	0	26.67	6.67	33.33	20	45	6.67	30	0	35	6.67	45	13.33	F
Ceratopogonidae	33.33	6.67	46.67	6.67	33.33	6.67	33.33	40	45	6.67	20	6.67	30	6.67	40	0	F
Tabanidae	26.67	20	13.33	6.67	33.33	6.67	26.67	26.67	25	0	15	6.67	30	6.67	20	6.67	F
Stratiomyiidae	53.33	13.33	40	0	40	6.67	60	6.67	55	20	20	0	70	13.33	70	20	F
Xylophagidae	46.67	6.67	6.67	0	40	0	0	0	/	/	/	/	/	/	/	/	F
Rhagionidae	6.67	6.67	26.67	0	13.33	0	33.33	13.33	20	6.67	20	0	20	0	35	6.67	F
Empididae	33.33	6.67	53.33	0	53.33	0	26.67	6.67	30	6.67	30	0	40	6.67	20	0	F
Muscidae	280	60	360	93.33	340	106.67	433.33	133.33	230	66.67	280	60	360	80	265	53.33	S
Sphaeroceridae	20	0	40	6.67	26.67	6.67	20	0	20	0	40	0	60	0	25	6. 67	S
Milichiidae	20	0	26.67	0	33.33	6.67	20	0	/	/	/	/	/	/	/	/	S
Lonchaeidae	0	0	13.33	0	6.67	0	0	0	/	/	/	/	/	/	/	/	S
Platypezidae	33.33	0	33.33	13.33	33.33	20	53.33	26.67	20	0	35	13.33	30	13.33	60	26.67	S
Clusiidae	80	6.67	53.33	26.67	60	20	106.67	6.67	85	6. 67	45	26.7	80	26.67	55	26.67	S
Mesofauna & Microfauna																	
Onychiuridae	3340	633.33	3940	966.67	3946.67	906.67	4740	786.67	2480	733.33	3130	1020	3625	1106.67	3240	960	F
Neanridae	13.33	0	46.67	0	86.67	0	60	0	30	0	40	0	115	0	65	0	F
Hypogastruridae	280	106.67	293.33	146.67	340	180	246.67	166.67	340	106.67	235	106.67	280	86.67	255	93.33	H
Neelidae	226.67	6.67	606.67	20	473.33	26.67	366.67	13.33	180	46. 67	285	33.33	270	40	230	40	F
Sminthuridae	180	0	346.67	20	273.33	60	620	46.67	185	13.33	235	33.33	290	53.33	415	40	H
Isotomidae	3000	400	3806.67	546.67	3420	526.67	3186.67	466.67	1750	526.67	2570	686.67	2445	786.67	2585	713.33	F
Tomoceridae	406.67	0	173.33	0	146.67	6.67	506.67	0	200	0	245	0	310	0	410	0	F
Entomobryidae	453.34	13.33	726.67	20	593.33	66.67	566.67	66.67	485	46.67	595	60	540	60	540	107.34	F
Cyphoderidae	360	26.67	333.33	40	253.33	13.33	393.33	60	490	80	540	106.67	450	66.67	585	126.67	F
Paronellidae	213.33	0	273.33	13.33	206.67	33.33	513.33	13.33	275	33.33	285	60	205	40	295	46.67	F
Ologamasidae	233.33	0	406.67	13.33	346.67	13.33	320	20.	275	0	425	0	340	0	385	0	P
Ameroseiidae	93.33	0	306.67	6.67	553.33	13.33	326.67	6.67	170	0	280	0	315	0	280	0	P
Pachylaelapidae	300	6.67	226.67	13.33	393.33	6.67	273.33	6.67	395	53.33	340	100	530	86.67	390	60	P
Laelapidae	180	0	246.67	0	260	0	346.67	0	235	0	165	0	325	0	350	0	P
Phytoseiidae	86.67	26.67	333.33	26.67	333.33	26.67	186.67	60	50	6.67	295	80	350	80	285	100	P
Stibmaeidae	93.33	53.33	160	46.67	220	73.33	133.33	33.33	115	13.3	135	20	135	13.33	150	20	P
Erythraeidae	86.67	0	80	0	46.67	0	126.67	0	30	0	55	0	75	0	75	0	P
Cryptognathidae	220	33.33	193.33	40	253.33	46.67	253.33	100	215	80	190	40	305	93.33	385	146.67	F
Galumnidae	2446.67	466.67	2520	613.33	3133.33	493.33	2746.67	546.67	1600	700	2220	773.33	2370	960	2390	706.67	P
Oribatulidae	606.67	80	1013.33	126.67	1113.33	200	780	166.67	720	266.67	1055	353.33	880	413.33	1000	500	F
Liacaridae	533.33	93.33	873.33	146.67	986.67	266.67	1286.67	233.33	380	140	575	206.67	735	346.67	765	326.67	H
Oribotritiidae	146.67	20	160	13.33	140	13.33	113.33	20	110	6.67	75	20	75	6.67	75	13.33	F
Ameridae	6.67	0	60	0	73.33	0	53.33	0	/	/	/	/	/	/	/	/	S
Oppiidae	526.67	200	346.67	206.67	553.33	186.67	606.67	313.33	355	286.67	385	346.67	530	420	390	333.33	P
Nothridae	320	53.33	440	33.33	453.33	46.67	566.67	80	490	146.67	550	173.33	580	160	745	200	P
Hypochthoniidae	193.33	60	226.67	93.33	206.67	46.67	340	60	75	26.67	100	26.67	90	20	100	33.33	P
Lohmanniidae	286.67	60	580	66.67	300	66.67	420.	53.33	315	100	365	80	340	80	295	100	F
Archoplophoridae	100	0	126.67	6.67	193.33	6.67	146.67	0	70	0	90	0	90	0	110	0	F
Trombiculidae	100	53.33	106.67	73.33	120	46.67	220	113.33	115	100	85	73.33	100	80	155	113.33	S

continuation sheet

Cheyletidae	0	0	6.67	0	6.67	0	0	0	/	/	/	/	/	/	/	/	P
Tarsonemidae	126.67	13.33	166.67	20	220	26.67	206.67	20	175	26.67	125	13.33	170	13.33	145	20	H
Perlohmanniidae	146.67	0	220	0	413.33	0	440	0	65	0	125	0	150	0	150	0	F
Trombidiidae	173.33	0	120	0	140	0	300	0	85	0	135	0	100	0	115	0	H
Miccrodispidae	153.33	0	166.67	0	226.67	0	180	0	210	40	305	20	280	80	290	73.33	S
Pygmephoridae	266.67	20	160	13.33	140	13.33	420.	13.33	150	0	135	13.33	140	0	165	13.33	F
Gymnodamaeoidae	13.33	0	33.33	0	106.67	0	80	0	15	0	75	0	30	0	65	0	S
Alicoragiidae	60	0	160	0	113.33	0	193.33	0	120	0	150	0	130	0	210	0	H
Total individual	19626.67	3293.33	24646.67	4333.33	26446.67	4726.67	28380	4900	4353.33	20250	5340	22125	6186.67	22305	5826.67	4353.33	
Total group number	71	55	102	62	103	66	97	59	84	49	91	56	90	58	92	57	
